# The role of the MYD88-dependent pathway in MPTP-induced brain dopaminergic degeneration

**DOI:** 10.1186/1742-2094-8-137

**Published:** 2011-10-11

**Authors:** Janelle Drouin-Ouellet, Claire Gibrat, Mélanie Bousquet, Frédéric Calon, Jasna Kriz, Francesca Cicchetti

**Affiliations:** 1 Axe Neurosciences, Centre de Recherche du CHUL (CHUQ), T2-50, 2705 Boulevard Laurier, Québec, G1V 4G2, Canada; 2 Faculté de Pharmacie, Université Laval, 1050, avenue de la Médecine, Québec, G1V 0A6 Canada; 3 Département de Psychiatrie & Neurosciences, Université Laval, 1050, avenue de la Médecine, Québec, G1V 0A6 Canada

**Keywords:** MPTP, MyD88, Inflammation, Dopamine, Parkinson's disease

## Abstract

**Background:**

Mounting evidence supports a significant role of inflammation in Parkinson's disease (PD) pathophysiology, with several inflammatory pathways being suggested as playing a role in the dopaminergic degeneration seen in humans and animal models of the disease. These include tumor necrosis factor, prostaglandins and oxidative-related stress components. However, the role of innate immunity has not been established in PD.

**Methods:**

Based on the fact that the myeloid differentiation primary response gene (88) (MyD88) is the most common adaptor protein implicated in toll-like receptor (TLR) signaling, critical in the innate immune response, we undertook a study to investigate the potential contribution of this specific pathway to MPTP-induced brain dopaminergic degeneration using MyD88 knock out mice (MyD88-/-), following our observations that the MyD88-dependent pathway was critical for MPTP dopaminergic toxicity in the enteric nervous system. Post-mortem analyses assessing nigrostriatal dopaminergic degeneration and inflammation were performed using HPLC, western blots, autoradiography and immunofluorescence.

**Results:**

Our results demonstrate that MyD88-/- mice are as vulnerable to MPTP-induced dopamine and DOPAC striatal depletion as wild type mice. Furthermore, MyD88-/- mice show similar striatal dopamine transporter and tyrosine hydroxylase loss, as well as dopaminergic cell loss in the substantia nigra pars compacta in response to MPTP. To evaluate the extent of the inflammatory response created by the MPTP regimen utilized, we further performed bioluminescence imaging using TLR2-luc/gfp transgenic mice and microglial density analysis, which revealed a modest brain microglial response following MPTP. This was accompanied by a significant astrocytic reaction in the striatum, which was of similar magnitude both in wild type and MyD88-/- mice.

**Conclusions:**

Our results suggest that subacute MPTP-induced dopaminergic degeneration observed in the central nervous system is MyD88-independent, in contrast to our recent observations that this pathway, in the same cohort of animals, is critical in the loss of dopaminergic neurons in the enteric nervous system.

## Background

Parkinson's disease (PD) is a neurodegenerative disorder for which the mechanisms of neuronal degeneration are currently unclear. However, sustained neuroinflammation has been suggested to contribute to the pathophysiology of several disorders of the central nervous system (CNS), including PD. Indeed, evidence from a number of human post-mortem studies has revealed the presence of chronic neuroinflammation in PD patients [1,2]. Elevated levels of various inflammatory mediators such as tumor necrosis factor alpha (TNFα), interleukin (IL)-1β, IL-2, IL-6, interferon γ, inducible nitric oxide synthase (iNOS) and cyxlooxygenase-2 (COX-2), together with the presence of activated microglia and astrocytes, have all been observed in the brain of PD patients [3-15]. Furthermore, a reduced risk of developing the disease has been reported in individuals taking non-steroidal anti-inflammatory drugs [16-18].

Inflammation has also been shown to play a role in dopaminergic neurodegenerative processes in various animal models of PD. In the 6-hydroxydopamine (6-OHDA) model of PD, microglial activation [19-24] can be partially inhibited by minocycline, and this prevents neuronal degeneration [25]. Lipopolysaccharide (LPS) has also been shown to be a potent stimulator of glial cells in the CNS and to provoke the release of various cytokines and free radicals, leading to dopaminergic neuronal loss in the substantia nigra (SNpc) when injected intra-nigrally [24,26,27]. In the 1-methyl-4-phenyl-1, 2, 3, 6-tetrahydropyridine (MPTP) mouse model of PD, the activation of microglia in both the striatum and SNpc is well documented [28,29]. However, which specific inflammatory pathways are critically activated in response to MPTP is unresolved. For example, mice lacking both genes encoding for TNF receptors are completely protected against the decrease in striatal tyrosine hydroxylase (TH) and dopamine content following a single subcutaneous MPTP injection [30], seemingly consequential to the absence of microglial activation in these knock out (KO) mice [31]. The TNF pathway is activated by the release of the pro-inflammatory cytokine TNFα, which is associated with the acute phase of inflammation in reaction to MPTP [30] leading to the activation of Nuclear factor kappa B (NFκB), Mitogen-activated protein kinase (MAPK) or induced apoptosis. However, other inflammatory mediators have also been suggested to play a role in MPTP-induced dopaminergic degeneration, albeit not to the same extent. Mice depleted in gp91phox and iNOS are partially protected against MPTP-induced acute neurodegeneration [32-36]. Gp91phox is part of the membrane bound complex NADPH-oxidase which, upon activation, generates superoxide radicals [37]. iNOS is an enzyme that catalyzes the production of nitric oxide in response to pro-inflammatory cytokine production, which has been suggested to be involved in PD [38]. These two components are parts of the oxidative related stress pathways, which involve reactive oxygen species (ROS) production, contributing to tissue damage and death. Finally, mice depleted in COX-2 show incomplete nigral dopaminergic protection against MPTP-induced damages [39,40]. COX-2 is a rate-limiting enzyme which can be produced in response to an increase in pro-inflammatory cytokines and is responsible for the conversion of arachidonic acid into prostaglandins [41]. These pathways take also part in the innate immune response and can directly or indirectly activate the MAPK, p38 and NFκB pathways. However, they differ from the Myeloid differentiation primary response gene (88) (MyD88)-dependant pathway in that toll-like receptors (TLRs) have a wider range of specific pathogenous and endogenous ligands.

MyD88 is an adaptor protein required by all TLRs, with the possible exception of TLR3 [42]. TLRs are pattern recognition receptors and contribute to CNS neurotoxicity largely by initiating and regulating inflammatory activities [43-45]. In addition, the immune response to infectious and noninfectious pathologies in the CNS is prompted or amplified by the activation of certain TLRs by endogenous ligands [46,47]. TLR-induced signaling also activates the adaptive immune system, as testified by the secretion of type I interferon, which enhances dendritic cell maturation, activation of natural killer cells, antibody production, and differentiation of virus-specific cytotoxic T lymphocytes [44]. Furthermore, MyD88 is a TIR-domain-containing adaptor protein of the IL-1 receptor family [48]. The activation of the MyD88-dependent pathway leads to the production of different pro-inflammatory mediators via NFκB or p38 and Jun-N-terminal kinase (JNK) [49], while in contrast, the TRIF-dependent pathway, the most studied of MyD88-independent pathways, drives the induction of type I interferon as well as inflammatory cytokines and trophic factors [42].

While little is known about the role of MyD88 in neurodegenerative diseases, there is some evidence that it may be important. MyD88 present in bone-marrow hematopoietic cells has been demonstrated to be neuroprotective in an animal model of amyotrophic lateral sclerosis [50], although bone-marrow-derived cells lacking MyD88 improve Alzheimer's disease-like pathology in two different mouse models of the disease [51]. Observations on the possible involvement of the innate immune system in PD have only recently emerged. Increased expression of TLR4 and its co-receptor CD14 has been reported 14 days following a single MPTP injection in mice [52], along with induction of TLR3, TLR4, TLR9 and MyD88 following an acute MPTP treatment [53]. Furthermore, TLR4 has been shown to promote the clearance of α-synuclein and thus dopaminergic neuronal survival in a mouse model of multiple system atrophy characterized by oligodendroglial α-synuclein overexpression [54].

We have recently reported that the MyD88-dependent pathway is critical for dopaminergic neuronal degeneration induced by the neurotoxin MPTP in the enteric nervous system (ENS) of the mouse [55]. In the CNS, MPTP administration has repeatedly been used as a model for PD [56-58] and given our findings for the role of the MyD88-dependent pathway in the ENS [55], we have now sought to look at the contribution of this pathway to CNS dopaminergic cell loss using the same cohort of mice.

## Methods

### Animals and parkinsonian model

MyD88-deficient (MyD88-/-) mice exhibit a deficiency in T cell proliferation, induction of acute phase proteins and inflammatory cytokines in response to IL-1, along with the absence of interferon-γ production and natural killer cell activity in response to IL-18 [59]. They have also previously been shown to be unresponsive to LPS, a ligand of TLR4 [60]. Here, we used the MyD88-/- mouse because this protein is a paramount component of the innate immune response [42].

All animals (25-35 g) were acclimatized to standard laboratory conditions in a controlled-temperature environment maintained under a 12 h light/dark cycle with free access to food and water. All animal experiments were performed in accordance with the Canadian Guide for the Care and Use of Laboratory animals and all procedures were approved by the Institutional Animal Care Committee of Laval University. Adult C57BL/6 wild type (WT) and MyD88-/- mice maintained on a C57BL/6 background (n = 7-8 per group and time points) received seven intraperitoneal (i.p.) injections of freshly diluted MPTP-HCl (20 mg/kg; Sigma, St. Louis, MO, USA) dissolved in saline 0.9%. MPTP was administered in a subacute manner, twice a day at 12-hour (h) intervals on the first two days and once a day for three subsequent days [61]. Remaining animals received i.p. vehicle injections instead of MPTP administration. Mice were sacrificed 3 h and 14 days following the last MPTP injection, as previously described in our ENS study on this cohort of animals [55].

Transgenic TLR2-luc/gfp mice enabled us to study *in vivo *the microglial activation/TLR2 response to subacute MPTP treatment. These mice, maintained on a C57BL/6 background, bear a bicistronic DNA construct (reporter genes luciferase (luc) and green fluorescent protein (gfp)), which is under the transcriptional control of the murine TLR2 promoter [62]. Transgenic animals were identified by PCR detection of luciferase as previously described [62] and maintained as a heterozygous genotype. An additional group of C57BL/6 mice were also subjected to an identical MPTP and saline regimen and sacrificed 24 h, 7 days and 14 days following the end of the MPTP treatment. These animals were used for microglial density analysis and evaluation of striatal GFAP protein levels.

### In vivo bioluminescence imaging

TLR2-luc/gfp mice were scanned 1 h following MPTP injections (the 1^st ^to 4^th ^and 6^th^), as well as 3 h, 24 h and 7 days following the last injection. Animals were all sacrificed 14 days following the end of the MPTP treatment. The TLR2-luc/gfp mice were strictly used for imaging purposes. Images were collected using an IVIS^® ^200 Imaging System (CaliperLS-Xenogen, Alameda, CA, USA). Twenty minutes (min) prior to imaging sessions, mice were administered a luciferase substrate D-luciferine dissolved in saline 0.9% (150 mg/kg) (CaliperLS-Xenogen) via the i.p. route. Mice were anesthetized with 2% isoflurane in 100% O_2 _at a flow rate of 2 L/min and placed in a heated, light-tight imaging chamber. Images were collected according to a previously published protocol [62]. Briefly, bioluminescence imaging was performed using a high-sensitivity CCD camera with wavelengths ranging from 300 to 600 nm with different fields of views and an f/1 lens aperture. Exposure time for imaging was 2 min, bioluminescence emission was normalized and light output was quantified as the total number of photons emitted per second with the use of Living Image 4.0 acquisition and imaging software (CaliperLS-Xenogen).

### Tissue preparation for post-mortem analyses

Animals were sacrificed under deep anesthesia with ketamine/xylazine and perfused using a standard transcardiac infusion of PBS 1× (BioShop, Burlington, ON, Canada) containing protease (Sigma, St. Louis, MO, USA) and phosphatase inhibitors (sodium pyrophosphate 1 mM and 50 mM sodium fluoride). Brains were collected and either post-fixed in a solution containing 4% paraformaldehyde (PFA; pH 7.4) in PBS for 48 h and subsequently cryoprotected using a 20% sucrose solution or snap-frozen in 2-methyl-butane and then stored at -80°C. Post-fixed coronal brain sections of 25 μm thickness were cut using a freezing microtome (Leica Microsystems, Montreal, QC, Canada). Samples of the striatum were extracted for high performance liquid chromatography (HPLC) and western blot analyses, along with mesencephalon samples for the latter method. Coronal brain sections (12 μm) were cut on a cryostat and stored at -80°C for histological analyses.

### Dopamine and DOPAC HPLC quantification

Dopamine and 3, 4-dihydroxyphenylacetic acid (DOPAC) were measured by HPLC with electrochemical detection according to a previously published protocol [63] in WT and MyD88-/- mice sacrificed 14 days following the end of MPTP treatment. Extracts of striata were collected, and 200 μl of perchloric acid (0.1 N; Mallinckrodt Baker, Inc. Phillipsburg, NJ, USA) was added to generate a supernatant. Fifty μl of supernatant was then directly injected into the HPLC consisting of a 717 plus autosampler automatic injector, a 1525 binary pump equipped with an Atlantis dC18 (3 μl) column, a 2465 electrochemical detector, and a glassy carbon electrode (Waters Limited, Lachine, QC, Canada). The electrochemical potential was set at 10 nA. The mobile phase consisted of 47.8 mM NaH_2_PO_4_, 0.9 mM sodium octyl sulfate (Mallinckrodt Baker, Inc. Phillipsburg, NJ, USA), 0.4 mM EDTA, 2 mM NaCl and 8% MeOH (Mallinckrodt Baker, Inc. Phillipsburg, NJ, USA) at pH 2.9 and delivered at 0.8 ml/min. Peaks were identified using Breeze software (Waters limited, Lachine, QC, Canada). HPLC quantifications were normalized to protein concentrations. Protein measurements were determined with a bicinchoninic acid (BCA) protein assay kit (Pierce, Rockford, IL, USA) as described by the manufacturer's protocol.

### Histology and microscopy

Sections were processed for immunohistochemistry to visualize TH+ neurons of the SNpc. Sections were incubated for 30 min in 3% H_2_O_2 _and blocked in a 0.1 M PBS solution containing 0.1% Triton X-100 (Sigma, St. Louis, MO, USA) and 5% normal goat serum (NGS; Wisent, QC, Canada) for 30 min. After overnight incubation at 4°C with a rabbit anti-TH (1:5 000; Pel-freeze, Rogers, AR, USA), sections were washed in PBS and incubated for 1 h at room temperature (RT) in a PBS solution containing biotinylated goat anti-rabbit IgG (1:1 500; Vector Laboratories, Burlington, ON, Canada). After further washing in PBS, the sections were placed in a solution containing ABC (Elite kit; Vector Laboratories, Burlington, ON, Canada) for 1 h at RT. The bound peroxidase was revealed with nickel intensified DAB as the chromogen (Sigma-Aldrich, St. Louis, MO, USA) and 0.01% hydrogen peroxide in 0.05 M Tris (pH 7.6) at RT. The reaction was stopped after approximately 5 min by extensive washing in PBS. Following the Ni-DAB reaction, sections were dehydrated and coverslipped. Photomicrographs were taken with a Microfire 1.0 camera (Optronics, Goleta, CA) linked to an E800 Nikon microscope (Nikon Inc., Québec, QC, Canada) using the imaging software Picture Frame (Microbrightfield, Colchester, VT, USA) and prepared for illustration in Adobe Photoshop CS5 12.0 x32 and Adobe Illustrator CS5 15.0.0.

For microglial density assessment, immunofluorescence of nigral sections using an antibody against iba1 was performed. After overnight incubation at 4°C with a rabbit anti-iba1 (ionized calcium binding adaptor molecule 1; 1:1 000; Waco Pure Chemicals Industries, Richmond, VA, USA), sections were washed in PBS and incubated for 2 h at RT in a PBS solution containing the secondary antibody, goat Alexa Fluor 488-conjugated anti-rabbit (1:1 000; Invitrogen, Eugene, OR, USA). Following three washes in PBS, sections were placed in a solution containing DAPI (0.022%) for 7 min at RT and washed again twice before being mounted on slides and coverslipped using fluoromount (Southern Biotech, AL, USA) sealed with nail polish. Photomicrographs were taken using a fluorescent light microscope (Olympus Provis AX70, Melville, NY, USA).

### Stereological quantification

The density of TH+ neurons and iba1+ microglia was assessed in the SNpc. TH measurements were performed under bright-field illumination, while iba1+ cell counting was performed using fluorescent light. Four sections of a 4-section (time course of inflammatory events protocol) or 5-section series (protocol using MyD88-/- mice) [64] were sampled using the Stereo investigator software (MicroBrightfield, Colchester, VT, USA) attached to an E800 Nikon microscope (Nikon Canada Inc, Mississauga, ON, Canada). The optical fractionator method [65] was used for cell counting and volume measurements through a 20× objective with the following counting parameters: distance between counting frames (150 μm × 150 μm), counting frame size (100 μm) and guard zone thickness (2 μm). Cells were counted only if they did not intersect forbidden lines.

### [^125^I]-RTI-121 autoradiography

Dopamine transporter (DAT) binding was evaluated with [^125^I]-RTI-121 [3β-(4-[^125^I]-iodophenyl) tropane-2β-carboxylic acid isopropylester] (NEN-DuPont, Boston, MA, USA; 2200 Ci/mmol) at 14 days following the last MPTP injection according to a previously published protocol [66]. Briefly, cryostat tissue sections were preincubated at RT for 30 min in a phosphate buffer (10.1 mM NaHPO_4_, 1.8 mM KH_2_PO_4_, 137 mM NaCl, and 2 mM KCl pH 7.4). Dried sections were subsequently incubated for 90 min at RT with 20 pM [^125^I]-RTI-121. Nonspecific binding was determined in the presence of 10 μM mazindol (Novartis, Basel, Switzerland). Following two 20 min washes in phosphate buffer, sections were briefly rinsed in ice-cold distilled water. When dry, sections were apposed against BiomaxMR radioactive sensitive films for 24 h.

### Quantification of [^125^I]-RTI-121 autoradiography

Digitized images of the striatum were obtained with a CCD camera model XC-77 (Sony Electronics Inc., New York, NY, USA) equipped with a 60 mm f/2.8 D magnification lens (Nikon Canada Inc., Mississauga, ON, Canada). The optical density of [^125^I]-RTI-121 specific binding was analyzed on a MacIntosh computer using Image J software (NIH, http://rsbweb.nih.gov/ij/). The average labeling for each area was calculated from three adjacent brain sections of a 1/20 series of the same animal at the level of the striatum (AP levels: 1.25 mm to 0.53 mm) [64]. Non-specific binding was subtracted from every measurement.

### Sample preparation and western immunoblotting

Samples were homogenized in 8 volumes of lysis buffer (150 mM NaCl, 10 mM NaH_2_PO_4_, 1% triton X-100, 0.5% SDS, and 0.5% deoxycholate) containing a cocktail of protease and phosphatase inhibitors. Samples were sonicated (3 × 10 sec) and centrifuged at 100 000 × *g *for 20 min at 4°C. The supernatant was collected and stored at -80°C. The protein concentration was determined using a BCA protein assay kit with bovine serum albumin as the standard. Fifteen μg of total protein per sample was added to laemmli loading buffer and heated to 95°C for 5 min. Samples were then loaded and subjected to sodium dodecyl sulfate-polyacrylamide (8%) gel electrophoresis. Proteins were electroblotted onto 0.45 μm PVDF membranes (Immobilon, Millipore, MA, USA) and blocked in 5% nonfat dry milk and 1% BSA in PBS for 1 h. Membranes were immunoblotted with mouse anti-GFAP (1:10 000; Sigma, St. Louis, MO, USA), rabbit anti-TH (1:5 000; Pel-freeze, Rogers, AR, USA) or mouse anti-β-actin (1:10 000; Applied Biological Materials Inc., Richmond, BC, Canada) and HRP-conjugated goat anti-mouse and anti-rabbit secondary antibodies (1:100 000; Jackson Immunoresearch Inc., West Grove, PA, USA) followed by chemiluminescence reagents (KPL, Mandel scientific Inc., Guelph, ON, Canada). Band intensities were quantified using a KODAK Image Station 4000 Digital Imaging System (Molecular Imaging Software version 4.0.5f7, KODAK, New Haven, CT) or a ImageQuant LAS 4000 luminescent image analyzer (GE Healthcare, Piscataway, NJ, USA).

### Statistical analyses

Statistical analyses were performed using PRISM 4 (Graphpad Software, San Diego, CA, USA) and JMP software 6.0.2 (SAS Institute Inc., Cary, IL, USA). All data derived from the animal experiment analyses are expressed as group means ± S.E.M. Unless otherwise stated, statistical analyses were performed using a one-way ANOVA and a two-way ANOVA to detect possible effects of MPTP treatment and genotype or time of sacrifice. Statistical significance was determined at an alpha level of 0.05.

## Results

### MyD88 depletion does not prevent MPTP-induced striatal dopaminergic degeneration

**Figure 1:**
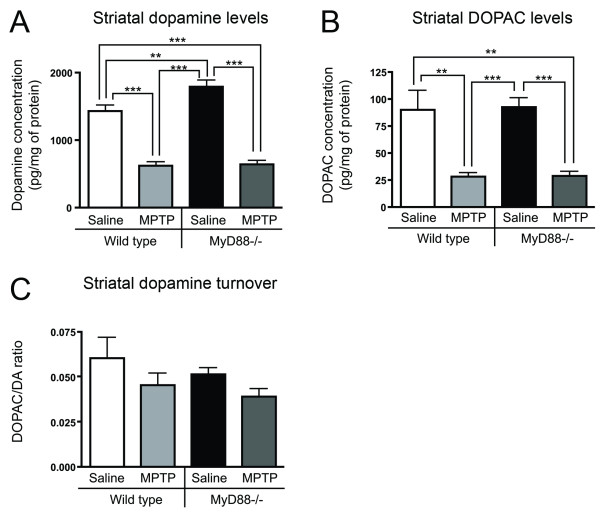
**MyD88-/- mice display striatal MPTP-induced dopaminergic depletion**. (**A**) Striatal measurements of dopamine levels, as performed by HPLC coupled to electrochemical detection, showing a decrease in dopamine content in WT (p < 0.001) and MyD88-/- (p < 0.001) MPTP-injected mice, as compared to saline-treated WT and MyD88-/- mice. (**B**) Similar results were obtained for DOPAC levels (p < 0.01 for WT and p < 0.001 for MyD88-/- vs. saline-treated respective controls). (**C**) Striatal dopaminergic turnover did not differ across groups. Statistical analyses were performed using one-way ANOVA. **p < 0.01, ***p < 0.001.

Striatal dopamine and DOPAC contents were measured in WT and MyD88-/- to evaluate the role of the MyD88-dependent pathway in MPTP-induced striatal dopaminergic depletion 14 days following the last MPTP injection. WT mice subjected to the subacute MPTP protocol had a 56.6% decrease in dopamine content (p < 0.001) as compared to the WT saline-treated group, and MPTP-injected MyD88-/- mice showed a similar 64.5% decrease (p < 0.001) compared to MyD88-/- saline-treated animals (Figure 1A). Although saline MyD88-/- expressed significantly higher striatal basal dopamine levels as compared to saline WT mice (20.2%; p < 0.01), they did not show exacerbated dopamine depletion as compared to MPTP-treated WT. MyD88-/- mice did not display different basal DOPAC levels compared to WT. In addition, both MyD88-/- and WT mice expressed identical levels of DOPAC depletion following MPTP administration, with a 69.2% decrease between both WT saline- and MPTP-treated mice (p < 0.01) and MyD88-/- saline- vs. MPTP-treated mice (p < 0.001; Figure 1B). No change was observed in striatal dopaminergic turnover across groups (Figure 1C).

**Figure 2:**
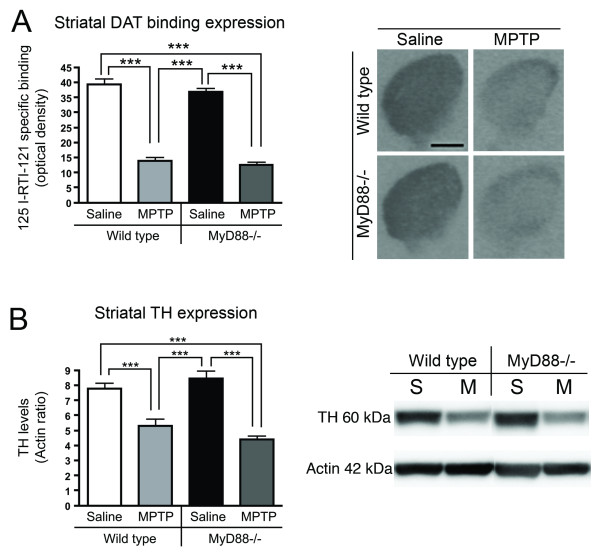
**MyD88-/- mice have MPTP-induced dopaminergic striatal terminal degeneration**. (**A**) Striatal measurements of DAT binding, as assessed by [^125^I]-RTI-121 autoradiography, revealed a significant decrease both in WT and MyD88-/- mice, as compared to vehicle treated WT and MyD88-/- mice (p < 0.001). (**B**) Western blot analysis of TH protein expression in the striatum further demonstrated a loss of dopaminergic fibers in both MPTP-treated groups (p < 0.001). Statistical analyses were performed using one-way ANOVA. ***p < 0.001. Scale bar in **A **= 1 mm. Abbreviations: S: saline-treated; M: MPTP-treated.

We further assessed whether the MyD88-dependent pathway was involved in the MPTP-induced degeneration of striatal dopaminergic terminals via the quantification of DAT binding and TH protein expression. [^125^I]-RTI-121 binding levels were diminished to the same level in MPTP-treated WT and MyD88-/- mice, as compared to their respective saline-injected controls at 14 days following the end of the MPTP challenge (68.1%; p < 0.001 and 65.8%; p < 0.001, respectively; Figure 2A). Analysis of DAT levels was further performed by dividing the striatum into 4 quadrants (dorsomedial, dorsolateral, ventromedial, and ventrolateral), which yielded similar results (data not shown). Comparable results were obtained for striatal TH protein expression. While MPTP-treated WT mice exhibited a 31.8% (p < 0.001) decrease when compared to their saline counterparts, a 47.8% (p < 0.001) reduction was observed between saline treated and toxin treated MyD88-/- mice (Figure 2B).

### The absence of MyD88 does not modify MPTP-induced nigral dopaminergic degeneration

**Figure 3:**
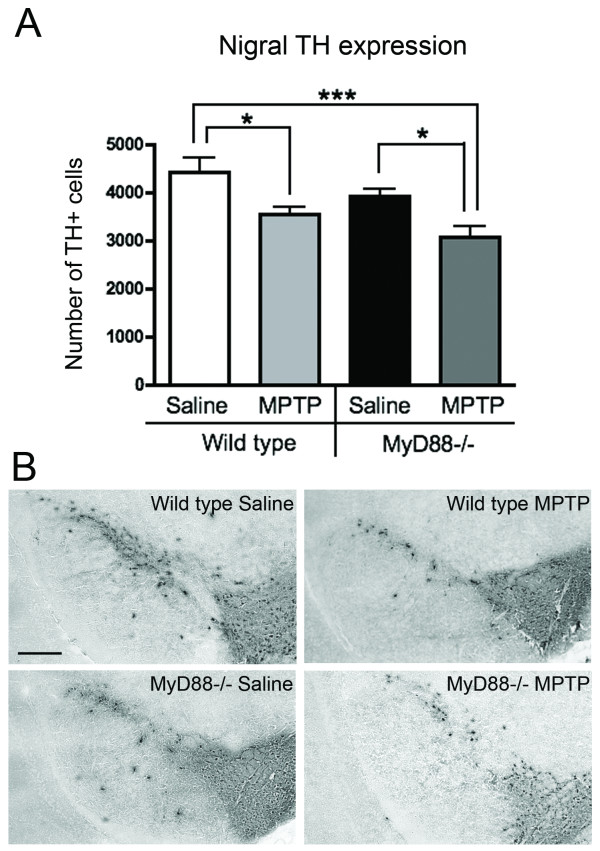
**Dopaminergic neurons are not protected from MPTP in MyD88-/- mice**. (**A**) Stereological counts of TH+ cells in the SNpc showed a decrease in dopaminergic neuronal count in WT mice that received MPTP, as compared to saline-treated WT mice (p < 0.05). MyD88-/- mice treated with MPTP were as vulnerable as WT, displaying a similar loss of dopaminergic neurons (p < 0.05). (**B**) Photomicrographs of TH immunohistochemistry staining of saline- and MPTP-treated animals at the level of SNpc. Statistical analyses were performed using one-way ANOVA. *p < 0.05, ***p < 0.001. Scale bar in **B **= 300 μm.

We next examined the role of the MyD88-dependent pathway on nigral TH+ cell bodies. No significant dopaminergic neuronal degeneration was observed 3 h following the end of the MPTP challenge, as revealed by stereological counts of SNpc TH+ neurons (data not shown). In contrast, WT animals, 14 days following the MPTP challenge, had a significant reduction in dopaminergic cell number in the SNpc, which was of similar magnitude between WT (20.5%; p < 0.05) and MyD88-/- (21.5%; p < 0.05) mice when compared to their respective saline-treated controls (Figure 3A, B). A two-way ANOVA revealed a significant effect of the MyD88-/- genotype in relation to the decrease in number of SN TH+ cells 14 days (p = 0.0293), but not at 3 h following the completion of the MPTP protocol (data not shown).

### Microglial response following the subacute MPTP challenge in WT mice

**Figure 4:**
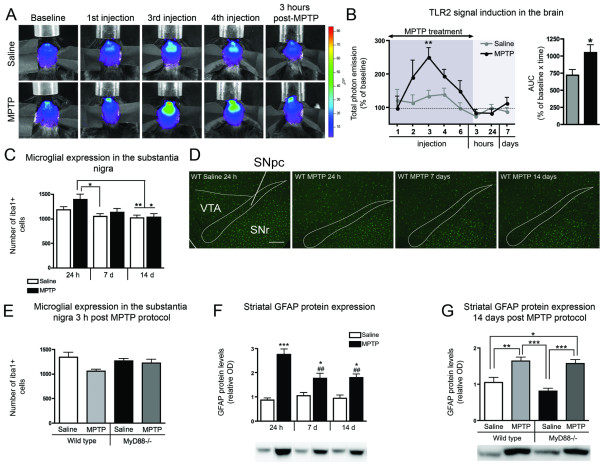
**Glial response in the subacute MPTP animal model and in MyD88-/- mice**. (**A**) Real-time imaging of the TLR2 response during the course of MPTP injections and 14 days following the last injection. Representative photographs of a saline- and an MPTP-treated mouse across time, showing significant brain TLR2 signal following the third MPTP injection, as compared to saline. The data is illustrated using pseudocolor images representing light intensity as indicated by the color scale, which were superimposed over gray-scale reference photographs. (**B**) (Left graph) Quantification of the total photon emission in the brain of saline- and MPTP-treated TLR2-luc/gfp mice at each time point assessed. (Right graph) Quantification of the areas under the curves in the two groups of animals. Statistical analyses were performed using student's *t*-tests. (**C**) Stereological count of iba1+ cells in the SNpc revealed a trend towards increased microglial expression in MPTP-treated mice 24 h following the last injection, which was significantly different from saline-treated animals at 7 (p < 0.05) and 14 days (p < 0.01), and from MPTP-treated mice at 14 days following the end of treatment (p < 0.05). (**D**) Photomicrograph of iba1 immunofluorescence staining of saline- and MPTP-treated animals at the level of SNpc, which has been outlined with a solid line. (**E**) 3 h following the last MPTP injection, similar microglial counts in the SNpc were seen in both saline- and MPTP-injected WT and MyD88-/- mice. (**F**) Western blot analysis of striatal GFAP protein levels revealed a strong increase in the MPTP-treated mice 24 h following the last injection, which had declined significantly 14 days following the end of treatment (p < 0.01) but still remained significantly increased compared to saline-controls at the 7-(p < 0.05) and 14-day time point (p < 0.05). (**G**) MPTP treatment induced a significant increase in GFAP striatal expression of similar magnitude in both WT (p < 0.01) and MyD88-/- mice (p < 0.001). Statistical analyses were performed using one-way ANOVA. *p < 0.05, **p < 0.01, ***p < 0.001 in comparison to saline-controls; ^##^p < 0.01 in comparison to MPTP-treated mice sacrificed at 24 h. Scale bar in **D **= 150 μm. Abbreviation: AUC: areas under curves; SNpc: substantia nigra pars compacta; SNr: substantia nigra reticulata; VTA: ventral tegmental area; WT: wild type.

The microglial response generated by the subacute MPTP regimen employed in the present study has not been as extensively characterized as in other MPTP lesioning protocols [32,67]. In order to assess the microglial activation during the course of MPTP injections and neuronal degeneration in real time, we capitalized on the TLR2-luc/gfp reporter mouse, a model in which microglial activation/innate immune response can be longitudinally visualized using biophotonic/bioluminescence imaging and high sensitivity/high resolution CCD camera [62]. The TLR2-luc/gfp mice were thus subjected to the subacute MPTP treatment, and microglial activation/induction of the TLR2 signal was quantified over time using *in vivo *imaging. A significant 1.9-fold increase in the TLR2 signal was observed following the third MPTP injection (p < 0.01) as compared to saline-treated controls, a response that returned to control values from the fourth injection onwards (Figure 4A, B). A subgroup of WT mice was sacrificed at 24 h, 7 and 14 days following the end of toxin administration. Stereological counts of iba1+ microglia in the SNpc revealed that, while MPTP-treated animals had a tendency towards increased iba1+ cell density in the SNpc at 24 h compared to saline-controls sacrificed at the same time (15.0%; p = 0.1), they had a significant increase in comparison to saline-controls at 7 (25.0%; p < 0.05) and 14 days (31.8%; p < 0.01) and MPTP-treated mice at 14 days (28.7%; p < 0.05). Two-way ANOVA revealed a significant effect of the time of sacrifice on microglial density (p = 0.0007), which confirms a reduction of iba1+ cell density across time in both saline and MPTP treated animals (Figure 4C, D). We subsequently assessed whether this slight increase in microglial density could be present at 3 h following the end of MPTP treatment. In WT and MyD88-/- mice sacrificed at 3 h, however, no change in microglial density was observed (Figure 4E), but an effect of MPTP treatment was revealed by a two-way ANOVA indicating a diminished iba1+ cell density in MPTP-treated groups.

### Astrocytic response following the subacute MPTP challenge

In the acute MPTP model, the astrocytic response has also been reported to play an important role in the events leading to dopaminergic degeneration [68], a response which seemingly occurs following microgliosis [67]. We thus investigated the astrocytic response at 24 h, 7 and 14 days following the completion of the MPTP challenge. Striatal GFAP protein quantification revealed substantially elevated levels at the three time points analyzed (24 h, 7 days and 14 days following the end of MPTP treatment) in comparison to saline-controls sacrificed at the same time (68.5%: p < 0.001; 40.7%; p < 0.05; 47.1%; p < 0.05, compared to their saline-controls, respectively) (Figure 4F). Elevated GFAP levels detected in MPTP groups was time-dependent, as the groups sacrificed at 7 and 14 days had significantly decreased levels at these times as compared to MPTP treated animals sacrificed 24 h following the end of treatment (p < 0.01) (Figure 4F). Given the presence of a persistent astrocytic response in the subacute model of MPTP, we next investigated the role of MyD88 in the astrocytic response at 14 days following the completion of MPTP challenge. At this time point, MPTP-treated WT mice displayed a 36.1% increase (p < 0.01) in GFAP protein levels in the striatum as compared to WT saline-treated mice (Figure 4G). Similarly, MyD88-/- mice administered MPTP had a 48.5% (p < 0.001) increase in GFAP protein expression as compared to MyD88-/- controls. In contrast, western blot analysis of GFAP levels in the mesencephalon did not reveal increased GFAP protein expression at this time of sacrifice (data not shown). Thus we have shown that there is an equivalent striatal astrocytic response in MPTP treated animals regardless of whether they express MyD88.

## Discussion

Our results indicate that mice lacking the adaptor protein MyD88, an important element of the innate immune response, are not protected against the degeneration of the dopaminergic nigrostriatal pathway induced by the subacute administration of MPTP.

**Figure 5:**
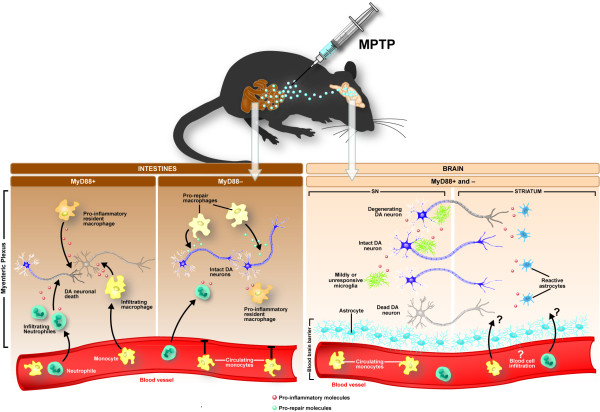
**Schematic drawing of the different role for MyD88 in the loss of dopaminergic neurons in the ENS vs. CNS following subacute MPTP challenge**. In the ENS, MPTP induces an immune response characterized by neutrophile and monocyte infiltration, and by a pro-inflammatory immunophenotype of resident macrophages. This immune response is accompanied by dopaminergic neuronal death. Depletion of MyD88 prevents macrophage infiltration and in fact promotes a shift to pro-repair immunophenotype in monocyte population of the ENS in response to MPTP. This favorably impacts the survival of the dopaminergic neurons in the myenteric plexus. In the brain of the same mice, however, dopaminergic neurons of the substantia nigra, along with striatal terminals, undergo degeneration/depletion when exposed to MPTP. These contrasting findings may relate to the type and intensity of the inflammatory responses provoked by MPTP in the two nervous systems. Another likely scenario relates to the BBB. Unlike in the ENS [55], cell infiltration from the blood to the brain may not occur following a subacute MPTP challenge. Abbreviation: SN: substantia nigra.

This result is of particular interest given that we have recently demonstrated that the dopaminergic neuronal toxicity induced by the same regime of MPTP administration in the myenteric plexus of the ENS **was **prevented by the absence of the MyD88-dependent pathway in this same cohort of animals [55]. The protective effect of MyD88 deletion was further linked to a switch in immunophenotype of the infiltrating macrophages from "M1" to "M2". Indeed, while "M1" polarized macrophages adopt pro-inflammatory functions, "M2" polarized macrophages coordinate tissue repair and remodeling, as well as immunoregulation [69]. This suggests that the MyD88-dependent pathway is implicated in the immunophenotype fate of macrophages contributing to the ENS inflammatory response induced by MPTP, in particular triggering the tissue repair functions of this cell type (see Figure 5) [55]. Based on the results obtained in the ENS using the subacute MPTP mouse model, we hypothesized that this pathway would have a similar role to play in dopaminergic neuronal degeneration in the CNS. Using the same cohort of lesioned mice, though, we failed to show an involvement of this pathway in the brain, at least within the nigrostriatal pathway. An obvious explanation for this difference in the role played by MyD88 in these two systems relates to the absence of a blood-brain-barrier in the gut, which potentially facilitates macrophage infiltration. In the CNS, however, the infiltration of macrophages has been observed in the acute MPTP mouse model of PD [70]. Differences in receptor expression between microglia and peripheral macrophages could also account for the absence of any effect of MyD88 in the CNS MPTP lesion. Although peripheral macrophages outside the CNS interact with other immune cells, such as T and B lymphocytes, microglia preferably interact with other CNS cells and this may be an important determinant as to the way in which MyD88 pathways cause neuronal cell loss [71,72]. Regardless, the contrasting role of the MyD88-dependent pathway in the ENS vs. the CNS raises the possibility that the pathophysiology of dopaminergic degeneration may be different from site to site, further suggesting that the use of anti-inflammatory agents targeting a specific pathway may not impact on neuronal losses found in PD.

Since the activation of microglia has been reported by various groups to be essential for MPTP-induced dopaminergic degeneration in the acute MPTP mouse model of PD [31,32,35], we assessed the extent of microgliosis at different time points during and following the end of MPTP treatment. Neuroinflammatory-induced microglial activation has been associated with a strong induction of several TLRs [45,73]. Despite low levels of TLR2 in the mouse brain in basal condition, it is strongly induced in microglia following infection or brain injury [62]. We therefore used bioluminescence imaging of the TLR2 response to evaluate *in vivo *the time course of the microglial response in our subacute MPTP model. Our results demonstrate an early and transient microglial response following the third MPTP injection, confirming that the MPTP regimen used in our study provides an adequate setting to study the MyD88-dependent pathway.

In accordance with the results obtained using bioluminescence imaging, no significant difference of microglial density was observed between the MPTP- and saline treated groups at any time point of sacrifice assessed following the end of MPTP treatment, although there was a significant decrease with time in both groups. This suggests that the MPTP mouse model utilized in this study is characterized by a much weaker microglial response, peaking at the 3^rd ^MPTP injection. MPTP-induced microglial activation has indeed been demonstrated to vary according to the regime employed. More specifically, it has been reported that acute MPTP injections (4 injections in 1 day) consistently induces microglial activation, whereas a more subchronic/subacute approach (1 injection per day for 5 consecutive days) triggers a significantly weaker response [74], similar to our observations using a 7 injection regime over 5-days of administration. This may help explain some of the discrepancies in the mechanisms leading to cell death outlined in the acute and subacute MPTP animal models. The acute model necessitates microglial activation while the subacute model is less dependent on this and relies for its toxic effects through the activation of molecules involved in the mitochondrial apoptotic cascade [75,76], such as caspase-11 [74], which also contributes to IL-1β secretion [77].

As mentioned above, several inflammatory pathways have already been shown to substantially contribute to acute MPTP-induced nigrostriatal degeneration, and include NFκB activation in astrocytes [78]. Based on our results, we cannot exclude the involvement of NFκB in MPTP-induced dopaminergic degeneration in the CNS, since while NFκB can be recruited by the MyD88-dependent pathway to produce pro-inflammatory cytokines [79], it can also be activated by MyD88-independent pathways.

In addition to the MPTP mouse model of PD, inflammation has been identified as a key player in other models using different toxins such as 6-OHDA and LPS. However, the stereotaxic disruption of the blood-brain barrier to generate both models allows for the infiltration of peripheral immune cells, an event for which the MyD88-dependent pathway has previously been identified to play a role in the MPTP-induced dopaminergic degeneration in the ENS [55]. In addition, while LPS, a TLR4 ligand, provides a suitable model to study the effects of activated microglia on a degenerating system (e.g. dopamine), it would not have been suitable for our study, given that MyD88-/- mice are unresponsive to the toxin [60].

Despite increased mRNA levels of TLR4, TLR9 and MyD88 in mice acutely treated with MPTP [53], our results do not suggest a fundamental role for the MyD88-dependent pathway in MPTP-induced dopaminergic degeneration, at least not in the CNS in the context when the toxin is administered subacutely. Because apoptosis is likely to play an important role in degenerating processes of the SNpc in the subacute MPTP model of PD used here, cellular-debris-derived damage associated molecular pattern molecules (DAMPs), as the result of cell death, could have generated a secondary inflammatory response driven by the MyD88-dependent pathway, creating additional dopaminergic death as observed, for example, in stroke models [80]. Our results, however, do not support this hypothesis, as no significant difference in the magnitude of CNS-related degeneration was seen between MPTP-treated WT and MyD88-/- mice.

## Conclusions

Our study provides important insights into the contribution of a paramount pathway of the innate immune system in mediating dopaminergic cell loss in the CNS in response to subchronic MPTP lesions. We have shown that while MyD88 is essential in the MPTP-induced dopaminergic cell death in the ENS, the nigrostriatal dopaminergic degeneration seen in the subacute MPTP mouse model of PD is MyD88-independent. These results highlight the dichotomy in the role of the MyD88-dependent pathway in the central vs. enteric nervous system. This sheds light onto the role of the inflammatory response as a whole to the pathophysiology of PD. Taken together, these findings have important implications for the development of novel therapeutic strategies to treat all aspects of PD.

## Competing interests

The authors declare that they have no competing interests.

## Authors' contributions

JDO participated in the design of the experiments, performed the animal studies, analyzed the data and wrote the manuscript. CG performed parts of the animal studies, and participated to tissue processing, the immunofluorescence and the GFAP western blot analysis. MB executed the HPLC analyses and parts of the western blot experiments. F. Calon provided resources for the HPLC analysis and revised the manuscript. JK provided the TLR2-luc/gfp mice and revised the manuscript. F. Cicchetti conceived the study, participated in its design and coordination and wrote the manuscript. All authors have read and approved the final version of the manuscript.
